# Wind speed prediction based on variational mode decomposition and advanced machine learning models in zaafarana, Egypt

**DOI:** 10.1038/s41598-025-98543-6

**Published:** 2025-05-04

**Authors:** Ali Taha, Nathalie Nazih, Peter Makeen

**Affiliations:** Electrical Engineering Department, Faculty of Engineering, The British University in Egypt (BUE), Al Shorouk City, Egypt

**Keywords:** Solar energy, Wind energy

## Abstract

Wind energy has become a key answer to the world’s energy problems, providing a clean and sustainable option instead of relying on fossil fuels. Enhancing wind energy systems and energy management is essential through efficient wind speed prediction. However, the complex nature of wind speed data contains significant challenges with existing forecasting models for long-term nonlinear forecasting accuracy, and this causes a lack of wind energy predictions, which may cause false distributions of energy. This study proposes a multi-step methodology that integrates Variational Mode Decomposition (VMD) with advanced machine learning like Extreme Gradient Boosting (XGBoost), Adaptive Boosting (AdaBoost), Light Gradient Boosting Machine (LightGBM), K-Nearest Neighbor (KNN), and transformer-based model (Informer) to improve long-term wind speed forecasting. The approach involves data collection from the NASA Power project, which consists of 35k samples of wind speed data, with performance evaluated on R-squared (R²) score and error metrics. The proposed approach demonstrated state-of-the-art performance, with LightGBM achieving the highest R² of 98% and the lowest error metrics. XGBoost and KNN performed slightly lower in R², achieving 97% score. Despite the high performance of the Informer model, it demonstrated the lowest in scores with a 78% R² score. The study’s novelty lies in highlighting the effectiveness and efficiency of VMD in addressing the complexities of wind speed data and underscores the potential of combining decomposition techniques with advanced machine learning models for accurate wind speed forecasting.

## Introduction

Improved technologies around the world cause an increased rate of fuel consumption, impacting the increased prices and high impact of CO2 emissions^1^. As the world faces the dual crises of resource depletion and environmental degradation caused by non-renewable energy consumption, wind power stands out for its affordability, abundance, and minimal ecological impact^2^. Wind energy, alongside solar power, is one of the most reliable renewable energy sources, capable of addressing the growing global energy demand driven by population growth and industrialization^3^. Wind energy has become a key answer to the world’s energy problems, providing a clean and sustainable option instead of relying on fossil fuels. As the world transitions toward cleaner energy systems, wind power is playing a pivotal role in mitigating climate change and ensuring a sustainable energy future^4^. Continued innovation in wind energy technology and forecasting methods will be vital to overcoming current limitations in the lack of wind energy predictions, which may cause false distributions of energy and managing demand difficulties^5^. However, challenges such as the complex nature of wind speed, along with limitations in meteorological data quality, hold back long-term forecasting accuracy^6–9^. Despite these challenges, advancements in forecasting techniques are crucial for balancing supply and demand in power grids and reducing reliance on fossil fuels^6–9^. Efficient wind speed forecasting is important in enhancing wind energy related systems, as it enhances energy management and grid stability^10^.

Recent studies have demonstrated the effectiveness of various decomposition techniques in handling the non-stationary nature of wind speed data. Sareen et al.^11^ combined k-nearest neighbor (KNN), Complete Ensemble Empirical Mode Decomposition with Adaptive Noise (CEEMDAN), and Bidirectional Long Short-Term Memory (BILSTM) for signal de-noising. When demonstrated on a dataset from the National Institute of Wind Energy (NIWE), their model achieved results with an R² of 94%, a Root Mean Squared Error (RMSE) of 0.41, and an Mean Absolute Error (MAE) of 0.31 for next-hour wind speed forecasting. Similarly, Bommidi et al.^2^ developed a hybrid model combining Improved Complete Ensemble Empirical Mode Decomposition with Adaptive Noise (ICEEMDAN) and a transformer model. When applied to wind farms in Block Island and Texas, their approach achieved remarkable results with an R² of 90% and an RMSE of 0.75 for 48-hour forecasts. Variational Mode Decomposition (VMD) combined with an autoencoder and optimized fuzzy cognitive mapping network is introduced in^6^. Their model demonstrated excellent performance for 144-hour forecasts when applied to wind speed data of three different coordinates in western mountains of Chongqing, China, with an R² of 98% and an MAE of 0.462. In a comparative analysis, Liang et al.^12^ evaluated seven different decomposition methods combined with Long Short-Term Memory (LSTM) for short-term forecasting, concluding that VMD outperformed other methods with an MAE of 0.086 and an RMSE of 0.112 for 5-hour predictions. The application of transformer architecture and deep learning models has shown promising results in wind speed forecasting. Wang et al.^10^ introduced a hybrid model combining random forest feature selection with a transformer for multi-step-ahead forecasting. Using National Renewable Energy Laboratory (NREL) data, their model achieved an MAE of 0.52 for 36-hour predictions, though with a moderate R² of 44%. Zhang et al.^13^ developed a more sophisticated approach using a multi-head attention-based probabilistic Convolutional Neural Networks (CNN) with a BiLSTM model, achieving an RMSE of 0.77 and an MAE of 0.56 for day-ahead forecasting at 2022 Winter Olympics venues. Lin et al.^14^ developed an adaptive spatiotemporal feature fusion transformer (GAOformer), performing with MAE 1.45 and Mean Squared Error (MSE) 3.85 for 2 h forecasting using data taken from the Fujian wind field. Several researchers have focused on developing models adapted to specific geographical and climatic conditions. In the Arctic region, Li et al.^15^ combined CEEMDAN with a CNN-LSTM model, achieving an MSE of 0.3960 and an RMSE of 0.6293 for 16-hour predictions using ERA5 reanalysis data. In Pakistan, Bashir et al.^3^ integrated decomposition techniques with Harris hawk’s optimization and a sequence-to-sequence model, achieving for 2-day predictions using World Bank data an RMSE of 0.639 and a MAE of 0.474. Chen et al.^4^ developed a spatial transfer-based hybrid model using CNN-LSTM-Autoencoder architecture for Chinese meteorological data, achieving impressive results with an MAE of 0.25 and an RMSE of 0.34 for 30-minute predictions. Recent research has explored innovative combinations of different methodologies. Jiang et al.^16^ integrated VMD, Graph Neural Networks, and Temporal Convolutional Networks for multi-step forecasting, establishing good results for R² and RMSE with 85% and 0.39, respectively, for 12-hour predictions using data from Shenzhen. Hilbert–Huang method with a nonlinear autoregressive dynamic neural network combined in^17^. Achieving prediction for 1 day ahead at Karamay wind farm for R² and RMSE with 90% and 1.99, respectively. Houndekindo et al.^18^ combined the gradient boosting algorithm in place of the random forest model. With the use of data from Environment and Climate Change in Canada (ECCC), achieved a next hour wind speed prediction MAE of 1.13 and RMSE of 1.47. Yu et al.^19^ maintained the combination of CNN with time-frequency recurrent neural network, performed an MAE of 1.32 and RMSE of 1.71 on 30-min wind speed forecasting with data obtained from the National Data Buoy Center. Particular attention has been paid to forecasting wind speed for a short-term period. Jiang et al.^20^ developed a Convolutional Gated Recurrent Unit network (CGRU) model with feature selection alongside secondary decomposition using Extreme Gradient Boosting (XGBoost), achieving an RMSE of 0.74 and an MAE of 0.53 for 2 h forecasting in Shandong Province. While focusing primarily on wind speed, some researchers have extended their work to wind energy applications. Yuan et al.^21^ combined an improved butterfly optimization algorithm with a relevance vector machine and Adaptive Boosting (AdaBoost) for short-term wind power prediction, achieving an R² of 95% and an RMSE of 10.403 for 15-minute predictions. Zeng et al.^22^ developed a Light Gradient-Boosting Machine (LightGBM) and Artificial Neural Network (ANN) hybrid model for wind power density forecasting across diverse terrains, achieving an average R² of 97% and an MAE of 10.55 for 1-hour forecasting. Al-Quraan et al.^23^ proposed a novel method for wind energy prediction in Jordan using the Whale Optimization Algorithm (WOA) to optimize parameters for Weibull, Gamma, and Rayleigh distribution models. The study evaluated wind energy potential across nine sites, achieving high accuracy with RMSE as low as 0.01013 and R² up to 0.98836. Al-Mhairat and Al-Quraan^24^ evaluated wind energy potential in Jordan using Weibull, Rayleigh, and Gamma distribution models, optimized with Particle Swarm Optimization (PSO), Grey Wolf Optimizer (GWO), and Whale Optimization Algorithm (WOA). The study found that the Gamma distribution combined with PSO (G-PSO) achieved the best performance, with RMSE as low as 0.00788 and R² up to 0.99777. Al-Quraan and Al-Mhairat^25^ compared different power models for calculating capacity factor and levelized cost of energy (LCoE) across multiple airport sites, with King Hussein Airport demonstrating the best results. The findings suggest that the exponential power model Q3(v) outperforms other models in terms of LCoE and capacity factor. Darwish and Al-Quraan^26^ utilized machine learning techniques to assess wind energy potential in Jordan, comparing the normal and Weibull probability distribution functions. Their study found that the normal PDF outperformed the Weibull PDF in estimating extractable wind energy, achieving improved accuracy through the application of 24 classifier algorithms. Stathopoulos et al.^27^ reviewed recent advancements in urban wind energy, focusing on wind resource assessment methods, including CFD and wind tunnel testing, and the integration of building-mounted wind turbines. The study emphasizes the need for further research in urban aerodynamics to optimize wind energy generation in urban environments. Al-Quraan et al.^28^ evaluated urban wind energy potential by comparing wind tunnel measurements with field data from two Montreal buildings, demonstrating less than 5% error in homogeneous terrain and up to 20% in non-homogeneous conditions, thus validating the wind tunnel approach for initial assessments.

Based on the literature review, various research studies demonstrated the concept of wind speed forecasting based on short-term and long-term forecast horizons using different machine learning models and decomposition methods in different areas, as summarized in Table 1. This table reveals the novelty of our paper compared with the current literature.

**Table 1:** Comparison between proposed method and previous research.

Author	Location	Methods	Period	*R*²	MSE	RMSE	MAE
Sareen et al.^11^	Gujarat, India	KNN + CEEMDAN + BiLSTM	1-hours	94%	NA	0.41	0.31
Bommidi et al.^2^	Block Island and Texas	Transformer + ICEEMDAN	48-hours	90%	NA	0.75	NA
Hu et al.^6^	western mountains of Chongqing, China	Autoencoder + VMD + Optimized fuzzy mapping network	144-hours	98%	NA	NA	0.462
Liang et al.^12^	Guangzhou	LSTM + VMD	5-hours	NA	NA	0.112	0.086
Wang et al.^10^	Denver	Transformer + RF feature selection	36-hours	44%			0.52
Zhang et al.^13^	China	CNN + BiLSTM	1-day	NA	NA	0.77	0.56
Lin et al.^14^	Fujian	GAOformer	2-hours	NA	3.85	NA	1.45
Li et al.^15^	Arctic region	CNN-LSTM + CEEMDAN	16-hours	NA	0.3960	0.6293	NA
Bashir et al.^3^	Pakistan	Seq-2-Seq + Harris hawk’s	2-days	NA	NA	0.639	0.474
Chen et al.^4^	China	CNN-LSTM-Autoencoder	30-minutes	NA	NA	0.34	0.25
Jiang et al.^16^	Shenzhen	Graph Neural Network + Temporal Convolutional Network + VMD	12-hours	85%	NA	0.39	NA
Chen et al.^17^	Karamay	Hilbert–Huang + Nonlinear Autoregressive Dynamic Neural Network	1-day	90%	NA	1.99	NA
Houndekindo et al.^18^	Canada	RF + Gradient Boosting	1-hour	NA	NA	1.47	1.13
Yu et al.^19^	Southern Mississippi	CNN + RNN	30-minutes	NA	NA	1.71	1.32
Jiang et al.^20^	Shandong Province, China	CGRU + XGBoost	2-hours	NA	NA	0.74	0.53
Yuan et al.^21^	NA	AdaBoost + Relevance Vector Machine	15-minutes	95%	NA	10.403	NA
Zeng et al.^22^	Average of Six Locations	LightGBM + ANN	1-hour	97%	NA	23.02	10.55
Proposed	Zaafarana, Egypt	LightGBM + VMD	1-month	98%	0.02	0.15	0.12

The contribution of this study is integrating VMD with advanced machine learning algorithms and transformer model to demonstrate accurate long-term forecasting of wind speed at 10 m using while accounting for the uncertainty and stochastic behavior of future wind speed. The study introduces a novel integration of VMD with machine learning and transformer models achieving lower errors than existing decomposition methods in Table 1 for long-term forecasting. The approach used in this study consists of multiple stages, beginning with the decomposition method, followed by hyperparameter tuning, and the implementation of machine learning and transformer models. Following this approach, the rest of the paper is organized as follows: Data and Methods presents the theory behind the algorithms used, describes the decomposition method, and explains the dataset employed. Results discuss the outcomes obtained from the proposed algorithms and compare them with previous studies. Finally, the conclusion summarizes the proposed methodology findings derived from it.

## System framework

**Fig. 1:**
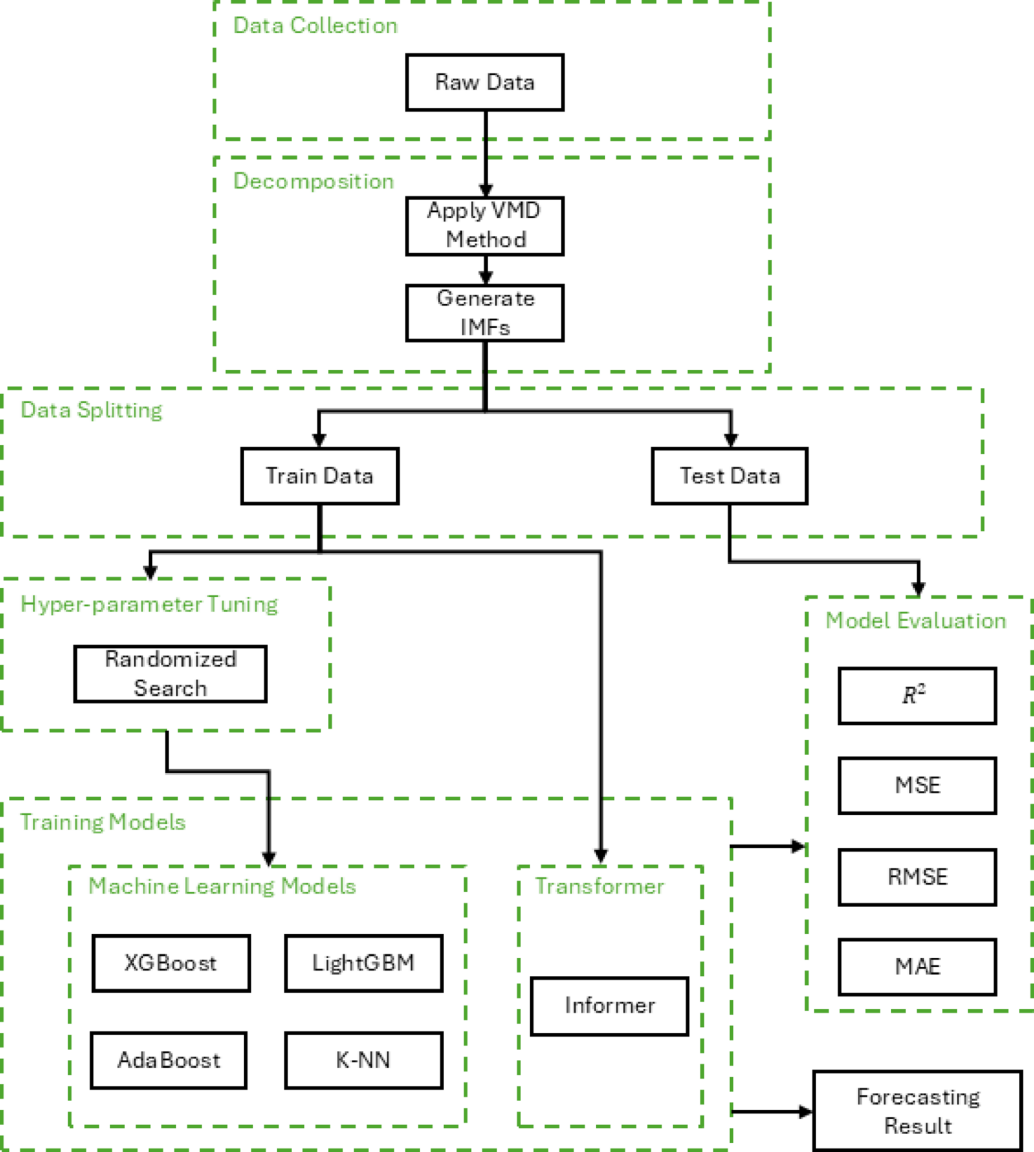
Framework of The Proposed Methodology.

The framework for wind speed forecasting involves a multi-step methodology that integrates data collection, feature decomposition, hyperparameters tuning and advanced machine learning techniques to achieve efficient accuracy for long-term forecasting. The proposed architecture is shown in Fig. 1.

1. **Data Collection**: Wind speed data at a height of 10 m is collected from the NASA Power project. This dataset serves as the foundation for the forecasting model, with wind speed selected as the target feature for prediction.
2. **Feature Decomposition**: To address the nonlinear and non-stationary characteristics of wind speed data, Variational Mode Decomposition (VMD) is applied. VMD decomposes the original wind speed signal into seven Intrinsic Mode Functions (IMFs), each representing a distinct frequency component of the data. These IMFs are used as the primary input features for the forecasting model, capturing the inherent complexity and variability of wind speed patterns.
3. **Hyperparameters Tuning**: To optimize the performance of machine learning models, a randomized search approach is employed for hyperparameter tuning. This method efficiently explores a wide range of hyperparameter combinations.
4. **Advanced Models Techniques**: The final stage involves training and evaluating machine learning and transformer-based models for long-term wind speed forecasting.

## Dataset and methodology

### System location and data compilation

**Fig. 2:**
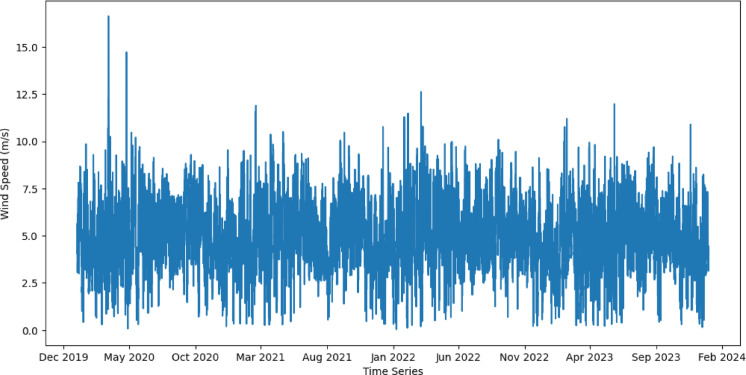
Wind Speed Original Data (January 1, 2020 – December 31, 2023).

The wind speed at the 10-meter dataset is sourced from the NASA power project^29^, which also provides comprehensive information about climate features like temperature, specific and relevant humidity, surface pressure, solar irradiance, and wind speed and direction at 10 m. Featuring a four-year timetable for data used for training from January 1, 2020 – December 31, 2023 with hourly intervals of wind speed measurements for accurate forecasting and a one-month timetable for testing data, Table 2 contains the details for the train and test data derived from the dataset. The dataset was taken at a specific location in Egypt called Al-Zaafarana wind park, which is considered the biggest wind farm in the Mideast^30^, with coordinates 29°12’00.0"N 32°36’00.0"E, the figure of the original data is shown in Fig. 2. This park contains a variety of characteristics, like an average wind speed of 10 m/s and geographic factors^30^. The data compilation has been followed by variational mode decomposition (VMD) method to gain robust and adaptive time-frequency analysis as will be illustrated in the following section.

**Table 2:** Wind speed dataset description for the region (January 1, 2020 – December 31, 2023).

Usage	Location	Interval	Duration		Samples	Max	Min	Mean
Train	Zaafarana, Egypt	1-hour	January 1, 2020	December 31, 2023	35,040	16.63	0.04	4.85
Test	Zaafarana, Egypt	1-hour	January 1, 2024	January 31, 2024	744	8.65	1.06	5.6

### Variational mode decomposition

The VMD algorithm begins with the idea of breaking a signal into a set of numbers of Intrinsic Mode Functions (IMFs). Each IMF has its own unique, slowly changing amplitude and frequency. The goal of the decomposition process is to identify the set of IMFs that most accurately capture the essence of the original signal. At the same time, the algorithm enforces certain constraints to ensure that the resulting IMFs are not only mathematically sound but also physically meaningful, making them useful for real-world applications^12^. The operation of the decomposition method happens on a variable principle, where the different measurements between the original and reconstructed signals are being minimized by the cost function produced by the method^12^. This process involves setting limits on each IMF’s amplitude and frequency. To solve this problem, a step-by-step approach is used to adjust the IMF values and the frequency and amplitude limits until an optimal solution is reached.

The VMD decomposes the original signal into a list of frequency bands, representing their characteristics as mode functions. The mode functions are collected by the optimization process to minimize the sum of squared Hilbert-transformed signal derivatives for each mode as shown in (1).

1 $$\:{min}_{\left\{{u}_{i}\right\},\left\{{\omega\:}_{i}\right\}}\left\{\sum\:_{i=1}^{L}{\left\|\left[{\partial\:}_{t}\left(\delta\:\left(t\right)+\:\frac{j}{\pi\:t}\right)*{c}_{i}\left(t\right)\right]{e}^{-j{\omega\:}_{i}t}\right\|}_{2}^{2}\:\right\}$$

2 $$\:x\left(t\right)=\:\sum\:_{i=1}^{L}{c}_{i}\left(t\right)$$

Where, $\:x\left(t\right)$ is the original signal, t presents time, and frequency bands as $\:{c}_{i}\left(t\right)$. The center frequencies are presented as $\:{\omega\:}_{i}=\:{\omega\:}_{1},\:\dots\:,\:{\omega\:}_{L}$. The Dirac function is denoted as $\:\delta\:\left(t\right)$ and $\:j$ as the imaginary unit. Where the center frequencies are specified to a frequency band.

## Advanced machine learning training models

### Extreme gradient boosting

Extreme Gradient Boosting (XGBoost) is a powerful and scalable machine learning algorithm that builds on the foundation of gradient boosting decision trees. It works by combining multiple classification and regression trees in a boosting framework. The core idea behind XGBoost is to iteratively improve the model by focusing on the errors made by the previous trees; each new tree is trained to correct the mistakes of the ones before it^20^.

To enhance its performance, XGBoost uses a second-order Taylor expansion to closely approximate the loss function, which improves precision. Additionally, it incorporates a regularization term into its objective function to control the model’s complexity. This helps prevent overfitting, ensuring that the model generalizes well with new, unseen data. These features make XGBoost a robust and efficient tool for both regression and classification tasks.

### Adaptive boosting

Adaptive Boosting (AdaBoost) dynamically adjusts the weights of the training samples during iterations, giving higher importance to those that were misclassified in previous rounds. This ensures that the algorithm focuses more on the harder to predict instances, allowing subsequent learners to prioritize and correct these errors^21^. This adaptive weighting mechanism is a key strength of AdaBoost, as it continuously refines the model’s focus on challenging data points, leading to improved accuracy over time. Ultimately, AdaBoost combines the predictions of all individual learners through a weighted voting system, where each learner’s contribution is based on its performance during training. This ensemble approach not only enhances the model’s robustness but also reduces the risk of overfitting, as it leverages the collective strength of multiple weak learners to produce a more accurate and reliable final prediction. By iteratively refining its focus and combining diverse learners, AdaBoost significantly improves the overall performance of the model, making it a powerful tool for complex prediction tasks.

### Light gradient-boosting machine

Light Gradient-Boosting Machine (LightGBM) is a highly efficient and scalable machine learning algorithm designed for gradient boosting frameworks. Unlike traditional gradient boosting methods that grow trees level-wise, LightGBM uses a novel technique called leaf-wise growth. This approach expands the tree by splitting the leaf that provides the largest gain in accuracy, resulting in faster training and often better performance^22^.

Another advantage of LightGBM is its support for parallel and distributed computing, allowing it to efficiently process massive datasets across multiple machines or cores. This scalability, combined with its accuracy and speed, has made LightGBM a popular choice for tasks like regression [18].

### K-Nearest neighbor

The k-nearest neighbors (k-NN) method relies on the idea of measuring the distance between two data points. To fill in missing values, it uses the average, either simple or weighted by distance, of the nearest observations. The choice of ‘k’ depends on the similarity of features. Finding the right value for ‘k’ is a key part of tuning the algorithm to improve its accuracy. Since there’s no straightforward way to determine the best ‘k’ we typically experiment with different values to find the most suitable one as shown in (3). Smaller values of ‘k’ can make the model sensitive to noise and outliers, leading to overfitting. On the other hand, larger values of ‘k’ create smoother decision boundaries, reducing variance but potentially increasing bias. Striking the right balance is crucial for achieving reliable results^11^. Therefore, the hyperparameter tuning method is used in this article, which will be talked about in the next sections. The k-NN equation is provided as follows:

3 $$\:{x}_{m}=\:\sum\:_{i}\frac{{c}_{i}{w}_{i}}{{w}_{i}};where\:{w}_{i}=\frac{1}{{d}_{i}}$$

where, the predicted value for the target point is presented as $\:{x}_{m}$. $\:{c}_{i}$ is the observed value of the i^th^ neighbor, and $\:{w}_{i}$ is the weight of the i^th^ neighbor, calculated as $\:{w}_{i}=\frac{1}{{d}_{i}}$. The distance between target and neighbor is $\:{d}_{i}$.

### Informer

**Fig. 3:**
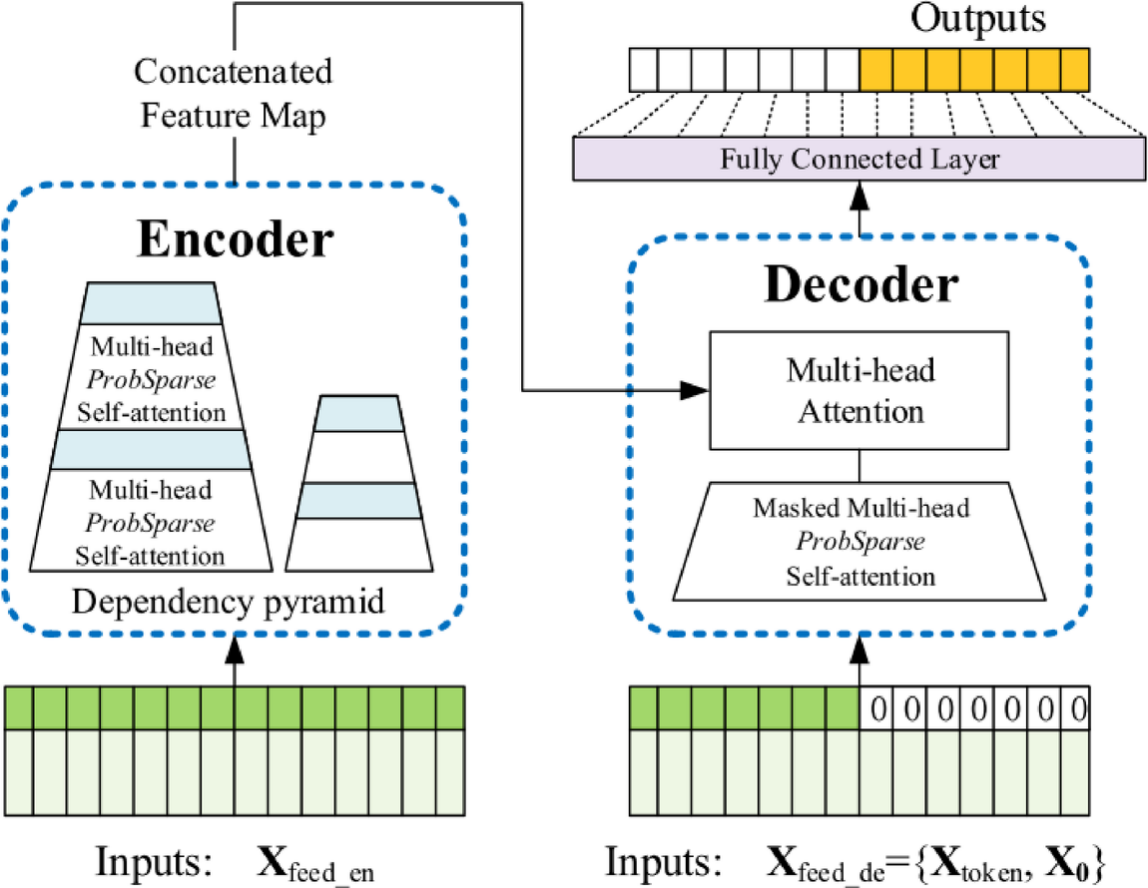
The Structure of The Informer Model^31^.

The Informer transformer model proposed by Zhou et al.^31^, Informer, is an advanced prediction algorithm that enhances the Transformer architecture for improved performance. It consists of an encoder and a decoder, where time series data are processed through the encoder. The time complexity is optimized by the algorithm with the use of ProbSparse self-attention. Also, a special process within the self-attention mechanism helps shorten the time-related part of the input sequence. Finally, the decoder generates output. Figure 3 illustrates the architecture of the transformer.

PropSparse self-attention allows each key to attend to the dominant queries through calculations using the scaled dot product as follows:

4 $$\:A\left(Q,K,V\right)=Softmax\left(\frac{Q{K}^{T}}{\sqrt{d}}\right)V$$

Q represents the sparse matrix, and d denotes the input sequence dimension. The product QK is used to determine the relationships or dependencies within the data. A SoftMax function is then applied to the aggregated data to compute attention scores, which indicate the importance of each location in the sequence^32^. The attention for the i^th^ query can be thought of as a kernel smoother expressed in probabilistic terms:

5 $$\:A\left({q}_{i},\:K,\:V\right)=\sum\:_{j}\frac{k({q}_{i},{k}_{j})}{{\sum\:}_{l}k({q}_{i},{k}_{l})}{V}_{j}=\:{\mathbb{E}}_{p\left({k}_{j}\right|{q}_{i})}\left[{V}_{j}\right]$$

where the asymmetric exponential kernel is selected by $\:k({q}_{i},{k}_{j})$, the probability $\:p\left({k}_{j}\right|{q}_{i})$ helps the self-attention on combining the values and acquires outputs. Quadratic time complexity dot product with $\:O\left({L}_{Q}{L}_{K}\right)$ memory usage is required for traditional self-attention. The i-th query vector sparsity measurement is evaluated using Kullback-Leibler divergence and the formula as follows:

6 $$\:M\left({q}_{i},\:K\right)=\:\text{ln}\sum\:_{j=1}^{{L}_{K}}{e}^{\frac{{q}_{i}{k}_{j}^{T}}{\sqrt{d}}}-\:\frac{1}{{L}_{K}}\:\sum\:_{j=1}^{{L}_{k}}\frac{{q}_{i}{k}_{j}^{T}}{\sqrt{d}}$$

The first term represents the Log-Sum-Exp of $\:{q}_{i}$ across all the keys, while the second term corresponds to their arithmetic meaning.

Encoder primary function is to understand relationships in long data sequences. The ProbSparse self-attention mechanism handles sequences that have extra V vectors. Consequently, the distillation operation assigns higher weights to the most important features in this scenario. The distillation procedure is as follows:

7 $$\:{X}_{j+1}^{t}=Maxpool\left(ELU\left(Conv1d\left({\left[{X}_{j}^{t}\right]}_{AB}\right)\right)\right)$$

Multi-head ProbSparse self-attention and the essential operations were contained by $\:{\left[.\right]}_{AB}$ representing the attention block, and $\:Conv1d(.)$ works as 1 dimensional convolutional filter with $\:ELU\left(.\right)$ As his activation function.

Decoder consists of two multi-head attention layers and takes input vectors as follows:

8 $$\:{X}_{{feed}_{de}}^{t}=Concat({X}_{token}^{t},\:{X}_{0}^{t})\epsilon{\mathbb{R}}^{\left({L}_{token}+{L}_{y}\right)\times\:{d}_{model}}$$

In this process, $\:{X}_{{feed}_{de}}^{t}$ represents the input to the decoder, $\:{X}_{token}^{t}$ serves as the start token of the sequence, $\:{X}_{0}^{t}$ acts as a placeholder for the target sequence. To maintain a consistent input dimension, the timestamps are padded with zeros. The masked multi-head attention mechanism makes sure that each part of the sequence pays attention only to the important information related to its own position, therefore avoiding self-regression. In the end, the final output is obtained.

## Evaluation metrics

To evaluate the effectiveness of the forecasting model, several metrics were used, like Mean Absolute Error (MAE), Mean Square Error (MSE), Root Mean Square Error (RMSE), and the coefficient of determination (R²), as represented from (9) to (12). MAE calculates the average absolute difference between the predicted values and the actual values, giving a clear and simple measure of how accurate the predictions are. MSE measures the average squared difference between predicted and actual values, while RMSE is the square root of MSE, providing a measure of error in the same units as the original data. R² was used to assess how well the model explains the variance in the data, with values closer to 1 indicating a better fit. These metrics collectively provide a comprehensive evaluation of the model’s forecasting accuracy and reliability.

9 $$\:MSE=\frac{1}{n}\sum\:_{i=1}^{n}{({y}_{i}-\widehat{{y}_{i}})}^{2}$$

10 $$\:MAE=\frac{1}{n}\sum\:_{i=1}^{n}|{y}_{i}-\widehat{{y}_{i}}|$$

11 $$\:RMSE=\sqrt{\frac{1}{n}\sum\:_{i=1}^{n}{({y}_{i}-\widehat{{y}_{i}})}^{2}}$$

12 $$\:{R}^{2}=\frac{SSR}{SST}=\frac{\sum\:_{i=1}^{N}{\left({y}_{i}-\widehat{{y}_{i}}\right)}^{2}}{\sum\:_{i=1}^{N}{({y}_{i}-\stackrel{-}{{y}_{i}})}^{2}}$$

where $\:{y}_{i}$ is the original wind speed at 10 m at $\:i$, $\:\widehat{{y}_{i}}$ is the forecasted wind speed number at $\:i$, $\:\stackrel{-}{{y}_{i}}$ is the actual wind speed average value at time $\:i$.

## Results and discussion

The methods were developed on an AMD Ryzen 5 4600 H with 16 GB RAMs and Nvidia GeForce GTX 1660 Ti GPU, and TensorFlow framework were used in python software programming with libraries like Sklearn, NumPy, Pandas, and any required library for the used methods.

### VMD implementation

**Fig. 4:**
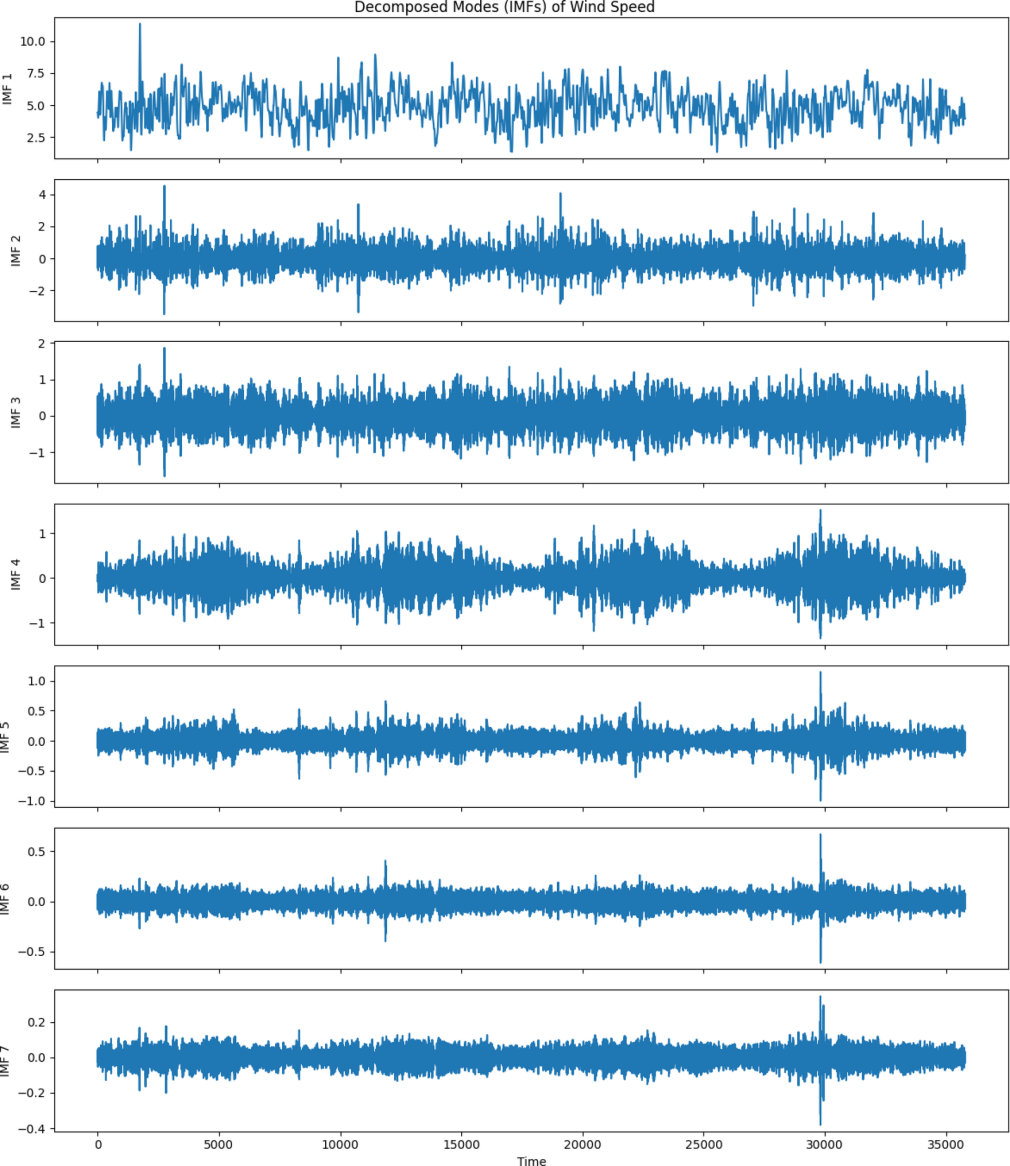
Frequency analysis of IMFs from VMD.

The VMD algorithm was applied to the wind speed data collected at a height of 10 m from the NASA Power project. As described earlier, VMD results in a limited number of IMFs from breaking down the original signal, each representing a different frequency and amplitude part of the wind speed signal. The wind speed time series data was preprocessed to remove any inconsistencies or missing values before being fed into the VMD algorithm. The algorithm was configured to decompose the signal into seven IMFs, as illustrated in Fig. 4. Each IMF represents a specific frequency band, ranging from high-frequency fluctuations to low-frequency trends, enabling a detailed analysis of the wind speed signal at multiple scales.

In the implementation of the VMD algorithm, several key parameters were configured to control the decomposition process. These parameters ensure the decomposition captures relevant frequency components while maintaining stability and accuracy. These parameters are described in Table 3.

**Table 3:** Parameters used in VMD.

Parameters	Value	Description
Alpha	2000	Controls the bandwidth constraint, moderating the separation between different modes
Tau	0	Enforces noise tolerance
K	7	Defines the number of distinct frequency components to extract from the wind speed signal
DC	0	Indicating that the decomposition excludes any direct current (DC) component from the modes.
Initialization	1	Specifying random initialization for the algorithm’s optimization process.
Tolerance	$\:{10}^{-6}$	Ensuring the algorithm stops when the difference between iterations becomes negligible

### Models configuration and Hyper-parameter tuning

In the proposed method, several machine learning models were employed to predict and analyze wind speed data efficiently. To optimize the performance of these models, hyperparameter tuning was performed using the Randomized Search method, a popular and efficient approach for exploring a wide range of hyperparameter combinations. However, for the Informer model, hyperparameter tuning using Randomized Search was not implemented. The Randomized Search method allows for quicker exploration compared to Grid Search, making it ideal for high-dimensional search spaces. The Randomized Search method was implemented to identify the best hyperparameters that maximize the models’ predictive performance. In Table 4, a detailed structures and parameters of each model after hyperparameter tuning.

**Table 4:** Models structure and hyperparameters values.

Model	Hyper-parameter	Value
XGBoost	Learning Rate	0.076
	Tree Depth	5
	Tree Count	271
	Child Weight	2
AdaBoost	Tree Depth	9
	Learning Rate	0.286
	Estimator Count	249
LightGBM	Learning Rate	0.075
	Max Depth	3
	Child Samples	21
	Estimator Count	394
KNN	Tree Size	49
	Neighbor Count	8
	Weighting Method	Distance
Informer	Encoder and Decoder Input Size	8
	Input Sequence Length	96
	Start Token Length	48
	Prediction Sequence Length	24
	Dimensional of Model	512

### Wind speed forecasting

To evaluate the effectiveness of the proposed approach and to highlight the impact of Variational Mode Decomposition (VMD) on the predictive models, we evaluated two scenarios: (1) models trained without VMD and (2) models trained with VMD-generated features. The results, summarized in Tables 5 and 6, illustrate a substantial improvement in forecasting accuracy with VMD.

1. Performance Without VMD Method.

**Fig. 5:**
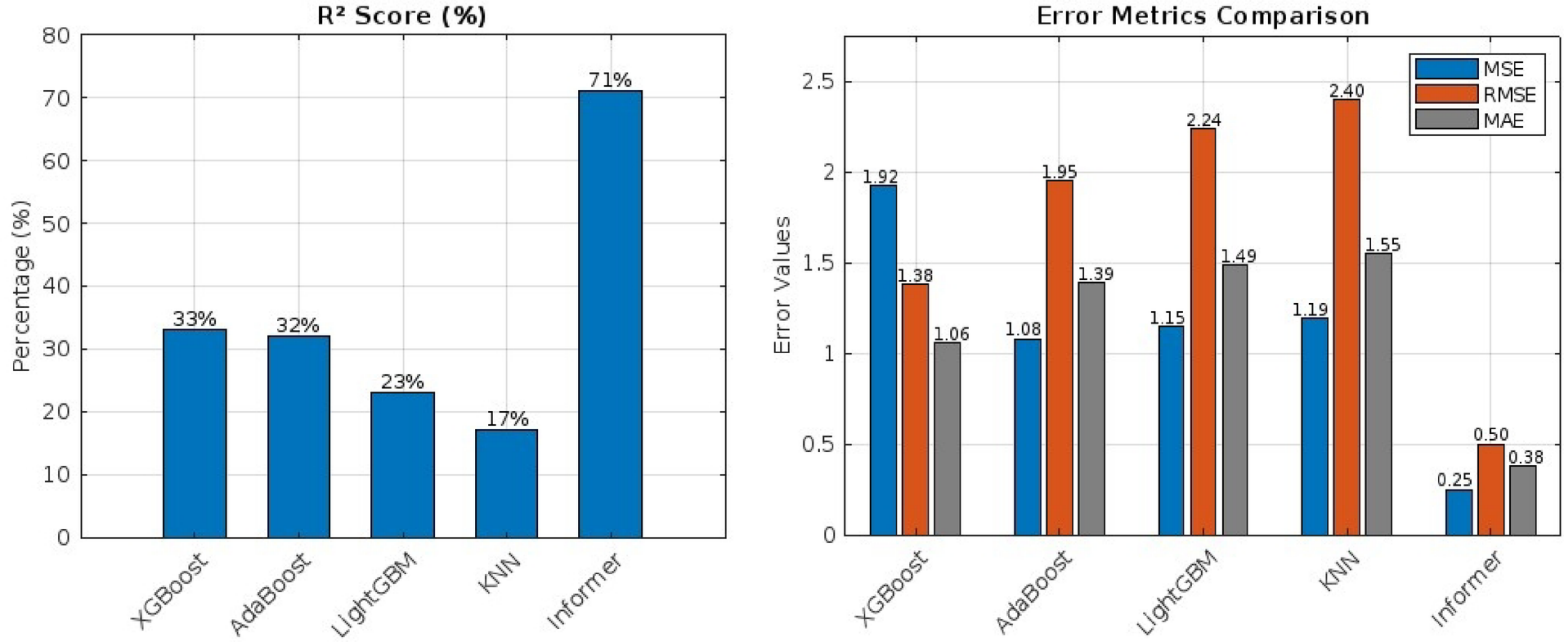
Performance without Using VMD Method (**a**) R², (**b**) MSE, RMSE, MAE.

In the first scenario, the models were trained using the traditional features extracted from the data source, such as year, month, day, hour, temperature, relative and specific humidity, surface pressure, solar irradiance, and wind direction at 10 m, with the wind speed at 10 m as the target feature. The performance metrics, including R², MSE, RMSE, and MAE, are summarized in Table 5. The Informer Transformer model achieved for R², MSE, RMSE, and MAE the best performance with 71%, 0.25, 0.5, and 0.38, respectively. Among the machine learning models, XGBoost performed relatively well for R², MSE, RMSE, and MAE of 33%, 1.92, 1.38, and 1.06, respectively, as shown in Fig. 5. However, the overall performance of the models in this scenario was limited, indicating challenges in capturing the complex patterns of wind speed using traditional features alone, but also showing the superiority of the Informer model in capturing long-term dependencies without the complexity of the data compared to other models.

**Table 5:** Performance without using VMD.

Models	*R*²	MSE	RMSE	MAE
XGBoost	33%	1.92	1.38	1.06
AdaBoost	32%	1.08	1.95	1.39
LightGBM	23%	1.15	2.24	1.49
KNN	17%	1.19	2.4	1.55
Informer	71%	0.25	0.5	0.38

2. Performance With VMD Method.

**Fig. 6:**
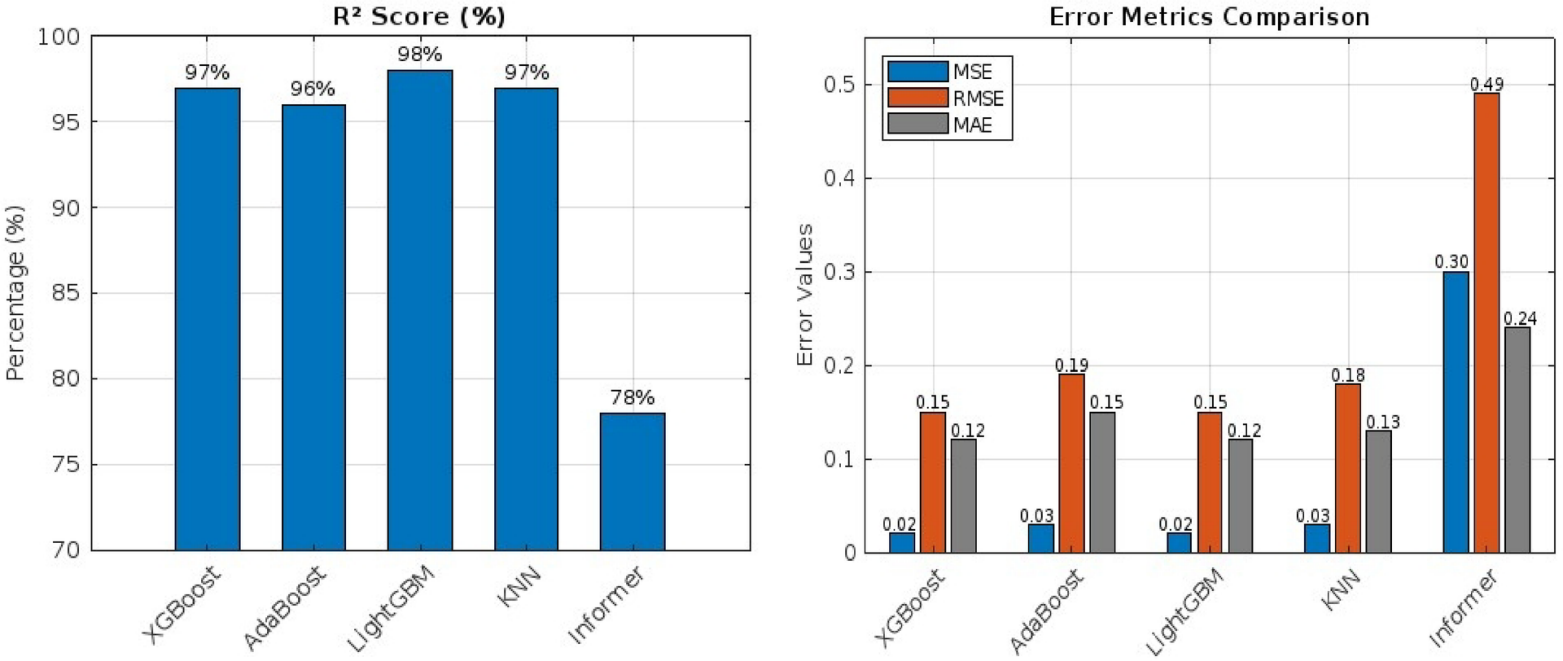
Performance with Using VMD Method (**a**) R², (**b**) MSE, RMSE, MAE.

In the second scenario, the Variational Mode Decomposition (VMD) method was applied to decompose the wind speed data at 10 m into seven Intrinsic Mode Functions (IMFs), which were then used as the primary input features. This approach significantly improved forecast performance across all models, as shown in Table 6. LightGBM achieved the highest R² of 98%, with an MSE of 0.02, an RMSE of 0.15, and an MAE of 0.12. XGBoost and KNN also demonstrated strong performance, with R² values of 97% for both, respectively. The transformer model, while still competitive, showed slightly lower performance in this scenario, with R², MSE, RMSE, and MAE of 78%, 0.3, 0.49, and 0.24, respectively, as shown in Fig. 6. These results highlight the effectiveness of VMD in enhancing the models’ ability to capture the underlying patterns of wind speed data.

**Table 6:** Performance with using VMD.

Models	*R*²	MSE	RMSE	MAE
XGBoost	97%	0.02	0.15	0.12
AdaBoost	96%	0.03	0.19	0.15
LightGBM	98%	0.02	0.15	0.12
KNN	97%	0.03	0.18	0.13
Informer	78%	0.3	0.49	0.24

The comparison between the two scenarios underscores the significant impact of the VMD method on forecasting accuracy. By decomposing the wind speed signal into its constituent IMFs, the models were able to achieve much higher R² values and lower error metrics compared to using traditional features alone. This improvement can be attributed to the ability of VMD to address the complex characteristics of wind speed data, providing the models with more meaningful and interpretable input features. The results demonstrate that VMD is a powerful preprocessing tool for wind speed forecasting, particularly when combined with machine learning and transformer-based models.

## Conclusion

This study has demonstrated the effectiveness of integrating Variational Mode Decomposition (VMD) with advanced machine learning and transformer-based models for long-term wind speed forecasting. By decomposing the wind speed signal into its constituent Intrinsic Mode Functions (IMFs), the models were able to capture the underlying patterns of wind speed data more effectively, leading to significant improvements in forecasting accuracy. LightGBM emerged as the top-performing model, achieving an R² of 98% and the lowest error metrics, an MSE of 0.02, when combined with VMD. The Informer transformer model also showed competitive performance, particularly in scenarios without VMD, highlighting its ability to capture complex dependencies in the data. The results underscore the importance of addressing the nonlinear and non-stationary characteristics of wind speed data through decomposition techniques like VMD. The limitation of this study is the dataset limited to a specific location and not varying in regions with different climate conditions, which might affect the performance of the models. Although the dataset time span is four years, incorporating a longer life span can enhance forecasting accuracy. Future research could explore the integration of additional meteorological variables instead of integrating the VMD method with only the wind speed variable and further refinement of transformer architectures to enhance forecasting accuracy by applying hyperparameters tuning method. Overall, this study contributes to the growing body of knowledge on wind speed forecasting and provides a robust framework for optimizing wind energy systems, ultimately supporting the global transition to sustainable energy sources.

## Data Availability

The dataset used during the current study is available online “https://power.larc.nasa.gov/“.
